# Predictors of persistence in patients with schizophrenia treated with aripiprazole once-monthly long-acting injection in the Spanish clinical practice: a retrospective, observational study

**DOI:** 10.1192/j.eurpsy.2021.23

**Published:** 2021-04-12

**Authors:** José Manuel Olivares, Ana González-Pinto, Mario Páramo

**Affiliations:** 1 Hospital Álvaro Cunqueiro, Complejo Hospitalario Universitario de Vigo, Vigo, Spain; 2 Hospital Universitario de Álava, Álava, Spain; 3 Complejo Hospitalario Universitario de Santiago Compostela, Santiago de Compostela, Spain

**Keywords:** schizophrenia, persistence, aripiprazole, antipsychotic, predictors

## Abstract

**Background:**

Poor adherence to antipsychotic drugs is a major problem in schizophrenia management and one of the most important risk factors for relapse and hospitalization. To date, there is little evidence on persistence predictors with long-acting injectable antipsychotics, especially with aripiprazole once-monthly (AOM). This study (NCT03130478) aimed to describe the impact of demographic and clinical characteristics on persistence with AOM treatment in real-world setting.

**Methods:**

This was an observational, retrospective, non-interventional study that included adult patients with schizophrenia who were initiated on AOM during a schizophrenia-related hospitalization. Data were retrospectively collected from patients’ medical records. The primary variable was persistence with AOM, measured as the number of days from AOM initiation up to all-cause AOM discontinuation during the first six months after treatment index.

**Results:**

140 patients were enrolled and 91 fulfilled the selection criteria. Six months after AOM initiation, 65 (71.4%) patients were still receiving AOM treatment, whereas 26 (28.6%) were not. The mean (standard deviation) time to AOM treatment discontinuation in the first six months was 138.1 (6.8) days, with most of the patients discontinuing at the first 28 days. The risk of AOM discontinuation in the first six months increases 1.05-fold annually since schizophrenia diagnosis (*p*=0.003); moreover, this risk increases 2.86-fold in patients with concomitant schizophrenia medication at AOM initiation compared to patients without concomitant schizophrenia treatments (*p*=0.02).

**Conclusions:**

Main factors predicting persistence with AOM treatment at six months in clinical practice are fewer years since schizophrenia diagnosis and not receiving concomitant schizophrenia treatments at AOM initiation.

## Introduction

Schizophrenia is a severe, chronic disabling disorder with most patients suffering repeated relapses [1–3]. Active psychotic episodes impact negatively on the illness course favoring disease progression and treatment refractoriness emergence, and preventing patients from recovering their previous functional and quality of life levels [4–6]. Similarly, very recent publications showed that duration of active psychotic symptoms after commencing treatment (DAT) strongly impacts long-term functional outcomes in schizophrenia [7]. Previous studies point the high relapse rate in schizophrenia, even after a single psychosis episode [1,8–11]. Hence, identifying main patient, disease and treatment factors contributing to lower adherence rates, as well as defining better strategies to improve treatment-adherence, is crucial in schizophrenia management.

Non-adherence to antipsychotic (AP) drugs is one of the most important risk factors for relapse and hospitalization [12–15]. Some systematic reviews reveal that almost 80% of patients are partially or totally non-adherent to oral AP [16,17]. Long-acting injectable (LAI) AP allow early non-adherence detection, facilitating corrective measures implementation to improve treatment adherence [18–20]. Despite meta-analyses of randomized controlled trials (RCTs) showed no advantages in relapse prevention associated with LAI AP in comparison with oral AP [21–26], those analyses closer to real clinical practice support clear evidence for LAI AP superiority on hospital admission prevention [27–31].

Contributing factors to AP treatment non-adherence had been previously studied [32–39], highlighting lack of insight, medication beliefs, side effects/tolerability issues, prior poor adherence and substance abuse as key drivers of non-adherence.

Aripiprazole once-monthly (AOM) is an atypical second-generation AP with proven efficacy and tolerability for schizophrenia treatment [40–45]. A mixed-treatment comparison of RCTs found lower AOM discontinuation rates due to adverse events (AEs) compared to other LAI AP [46], and recent studies support significant evidence in relapse prevention with AOM versus previous treatments [47,48]. However, few observational studies have assessed predictors of persistence with AOM treatment in patients with schizophrenia [49,50]. DOMINO study, which included 261 patients with schizophrenia, found higher treatment adherence among patients with baseline Clinical Global Impression (CGI) score < 5, lifetime schizophrenia dimension mania score < 6, and psychotic spectrum schizoid score < 12 [49]. Suzuki et al. (2018) [50] retrospectively analyzed 82 patients with schizophrenia in which AOM outpatient initiation and no-history of hospitalizations were significantly associated with lower AOM discontinuations.

In the present non-interventional study, we intend to evaluate the impact of patient demographic and clinical characteristics on AOM persistence (understood as time from treatment initiation up to treatment discontinuation for any reason) during the first six months of treatment in patients starting AOM after being stabilized from an acute psychotic relapse and prior to discharge following Spanish clinical practice.

## Methods

### Study design and participants

This was a multicenter, observational, retrospective, and non-interventional study that included adult patients (age ≥ 18 years) diagnosed with schizophrenia who were initiated on AOM treatment (before November 1^st^, 2016 and up to January 2015) during a schizophrenia-related hospitalization at least six months before data collection. Patients with a psychiatric disorder other than schizophrenia as primary diagnosis were excluded.

Study was approved by the participant sites (H.U Bellvitge, Numància Salut Mental, H. IAS Girona, Parc Sanitari.Sant Joan de Deu (St. Boi), Fundación Jiménez Díaz, H.G.U Gregorio Marañón, H. Infanta Leonor, H.U Virgen del Rocío, H.U.R Málaga, H. Álvaro Cunqueiro, H.U. Álava, CHU Santiago de Compostela, CAU de León, H. Ciudad Real) independent ethics committees, and patients gave their informed consent before inclusion, whenever possible. Patients deceased before study initiation or who could not be reached after reasonable efforts could also be included.

Index date was AOM treatment initiation date. Data were retrospectively collected from patients’ medical records, mainly from the index date until the last information available in the patient file at the time of data collection (minimum six months after index date). Data were collected from all visits occurring as per clinical practice during this period.

The following variables were collected: demographic characteristics (age, gender, marital status, education, and occupation), age of schizophrenia onset, number of previous schizophrenia relapses and of previous AP within the 2 years prior to the index date, concomitant schizophrenia treatments at index date, history of non-adherence in the 3 months prior to the index date, non-psychiatric and psychiatric comorbidities, living situation and family support, non-pharmacological interventions, alcohol and drug abuse/dependence, reason to initiate AOM treatment, hospitalization duration at index date, concomitant non-schizophrenia medications at index date, CGI-S score, Brief Psychiatric Rating Scale (BPRS) positive, negative and total scores, AOM treatment description, and AEs.

CGI-S is a 7-point scale (from 1 to 7; 1 represents less severe) that requires the clinician to rate the severity of the patient’s illness at the time of assessment [51].

BPRS evaluates severity of symptoms in patients with psychotic illness. It covers four symptom dimensions: anxiety and depression; positive symptoms; negative symptoms; and manic symptoms. BPRS total score ranges from 18 (no symptoms) to 126 (maximal severity) [52].

### Statistical analysis

The primary variable was persistence with AOM treatment during the first six months after treatment initiation. Persistence was defined as the time between index date and all-cause AOM discontinuation. Patient was discontinued if missed two consecutive or three non-consecutive AOM injections. Persistence was described by Kaplan–Meier means. Univariate analyses were performed to test the association between patients’ demographic and clinical characteristics and persistence with AOM treatment at six months. In addition, univariate and multivariate Cox Regression models were applied. The variables with *p* < 0.15 for the overall effect in the univariate Cox regression model were pre-selected. Collinearity was assessed between all pre-selected variables using spearman correlation for continuous variables, chi-square test or Fisher test for categorical variables and Whilcoxon–Mann–Whitney test for continuous variables crossed with categorical variables, as applicable. In case of a couple of variables strongly correlated (correlation coefficient > 0.6) or associated (*p* value < 0.05) only the one that brings most information to the model in the univariate cox analysis (with the smallest value of the “−2 LOG L” criterion) was pre-selected to be included in the model. Further refining of the pre-selected variables based on the amount of non-missing data as well as the clinical relevance of the studied variable.

The final multivariable model was obtained by using a “backward selection” method with the final set of selected variables. Only the variables with *p* < 0.05 in at least one category (or for the overall variable in case of continuous variables) were included. Estimated hazard ratios (HR) with 95% confidence intervals (CI) were calculated.

Analysis of the secondary objectives was descriptive. All collected data were presented using summary statistics.

Treatment patterns were described by changes in the schizophrenia therapy administered at index date (medication augmentations, switching and/or discontinuations, and reasons for each change). Switching was defined as initiation of an alternative schizophrenia medication (received for at least 30 days) to AOM before or within 30 days of this drug discontinuation. Medication augmentation was defined as initiation of a new schizophrenia medication not present at treatment initiation with a continuous overlap of at least 30 days with AOM.

Missing data were not replaced.

Statistical analyses were performed with the SAS statistical software package (SAS Institute, Inc, Cary, NC).

## Results

### Patients disposition, demographic data, and clinical characteristics

A total of 140 patients were enrolled in 15 Spanish sites, of which 91 fulfilled the eligibility criteria (Full Analysis Set [FAS] population). Of the FAS, 65 patients (71.4%) were defined as persistent in AOM treatment within the first six months after AOM initiation, whereas 26 (28.6%) were non-persistent.

In the overall population, 60 (65.9%) were males, with a mean (standard deviation [SD]) age of 39.8 (10.3) years at AOM initiation. Most of patients were unemployed both at AOM initiation and during the retrospective period (44.0 and 36.3%, respectively) and educated in secondary school (24.2%) (Table 1).

**Table 1.** Demographic data of included patients.

	Persistence at first six months	Non-persistence at first six months	Total
	(*N* = 65)	(*N* = 26)	(*N* = 91)
Age (years)—AOM initiation, mean ± SD	39.2 ± 10.5	41.2 ± 9.9	39.8 ± 10.3
Male gender, *n* (%)	42 (64.6)	18 (69.2)	60 (65.9)
Height (cm), mean ± SD	168.7 ± 9.5	171.4 ± 6.9	169.1 ± 9.2
Weight (kg)—AOM initiation, mean ± SD	81.0 ± 19.6	75.6 ± 7.0	80.3 ± 18.6
Weight (kg)—RP, mean ± SD	83.0 ± 19.8	75.9 ± 7.3	82.0 ± 18.7
Marital status—AOM initiation, *n* (%)			
Married	0 (0.0)	2 (7.7)	2 (2.2)
Living with a partner	5 (7.7)	0 (0.0)	5 (5.5)
Single	50 (76.9)	22 (84.6)	72 (79.1)
Divorced	9 (13.9)	1 (3.9)	10 (11.0)
Widow	1 (1.5)	0 (0.0)	1 (1.1)
Not available	0 (0.0)	1 (3.9)	1 (1.1)
Marital status—RP, *n* (%)			
Married	1 (1.5)	2 (7.7)	3 (3.3)
Living with a partner	5 (7.7)	1 (3.9)	6 (6.6)
Single	49 (75.4)	17 (65.4)	66 (72.5)
Divorced	9 (13.9)	1 (3.9)	10 (11.0)
Widow	1 (1.5)	0 (0.0)	1 (1.1)
Not available	0 (0.0)	1 (3.9)	1 (1.1)
Missing	0 (0.0)	4 (15.4)	4 (4.4)
Highest level of education—AOM initiation, *n* (%)			
No compulsory education	4 (6.2)	2 (7.7)	6 (6.6)
Compulsory education	15 (23.1)	5 (19.2)	20 (22.0)
Secondary school	17 (26.2)	5 (19.2)	22 (24.2)
High school	12 (18.5)	5 (19.2)	17 (18.7)
University degree	10 (15.4)	3 (11.5)	13 (14.3)
Not available	7 (10.8)	6 (23.1)	13 (14.3)
Highest level of education—RP, *n* (%)			
No compulsory education	4 (6.2)	1 (3.9)	5 (5.5)
Compulsory education	15 (23.1)	5 (19.2)	20 (22.0)
Secondary school	17 (26.2)	4 (15.4)	21 (23.1)
High school	12 (18.5)	4 (15.4)	16 (17.6)
University degree	10 (15.4)	2 (7.7)	12 (13.2)
Not available	7 (10.8)	10 (38.5)	17 (18.7)
Occupation—AOM initiation, *n* (%)			
Paid employment	13 (20.0)	3 (11.5)	16 (17.6)
Nonpaid activity	12 (18.5)	0 (0.0)	12 (13.2)
Student	5 (7.7)	3 (11.5)	8 (8.8)
Unemployed	25 (38.5)	15 (57.7)	40 (44.0)
Not available	10 (15.4)	5 (19.2)	15 (16.5)
Occupation—RP, *n* (%)			
Paid employment	14 (21.5)	5 (19.2)	19 (20.9)
Non-paid activity	13 (20.0)	0 (0.0)	13 (14.3)
Student	5 (7.7)	2 (7.7)	7 (7.7)
Unemployed	23 (35.4)	10 (38.5)	33 (36.3)
Not available	10 (15.4)	9 (34.6)	19 (20.9)
Living situation/family support—AOM initiation, *n* (%)			
Alone	18 (27.7)	6 (23.1)	24 (26.4)
With family or friends	43 (66.2)	18 (69.2)	61 (67.0)
Psychiatric institution	0 (0.0)	0 (0.0)	0 (0.0)
Sheltered accommodation	2 (3.1)	0 (0.0)	2 (2.2)
Other	2 (3.1)	1 (3.9)	3 (3.3)
Not available	0 (0.0)	1 (3.9)	1 (1.1)
Living situation/family support—RP, *n* (%)			
Alone	13 (20.0)	5 (19.2)	18 (19.8)
With family or friends	46 (70.8)	15 (57.7)	61 (67.0)
Psychiatric institution	2 (3.1)	0 (0.0)	2 (2.2)
Sheltered accommodation	2 (3.1)	0 (0.0)	2 (2.2)
Other	2 (3.1)	0 (0.0)	2 (2.2)
Not available	0 (0.0)	6 (23.1)	6 (6.6)

Abbreviations: AOM, aripiprazole once-monthly; RP, retrospective period; SD, standard deviation.

Mean (SD) time since schizophrenia diagnosis was significantly shorter among persistent patients compared to non-persistent ones (10.5 vs. 17.3 years; *p* = 0.01). For the overall population this value was 12.4 (10.3) years.

Patients had received a mean of 2.2 (1.7) previous AP within the 2 years prior to the date of AOM initiation, with increased numbers among persistent patients (2.4 vs. 1.7 [non-persistent]; *p* = 0.04). Similar tendencies were observed in the number of AP treatments received within the 5 years prior to index date; in that period, the most commonly administered AP treatment was aripiprazole (68.1%), followed by risperidone (52.8%), olanzapine (49.5%), and paliperidone (38.5%).

At index date, a higher percentage of persistent patients presented a history of non-adherence in the prior 3 months in contrast to the non-persistent population (43.1 vs. 30.8%).

Also remarkable are the main reasons to initiate AOM: “Prevent discontinuation” (83.5%), “Prevent relapse” (80.2%), and “Improve adherence to treatment” (76.9%). All these reasons were significantly more frequent in the persistent population (92.3, 87.7, and 84.6%) than in the non-persistent population (61.5, 61.5, and 57.7%; all comparisons *p* < 0.01). The “Patient preference” was also selected as a reason to initiate AOM in 13.9% of persistent patients, whereas none (0.0%) of the non-persistent patients reported this reason.

At index date, comorbidities were less common among persistent patients (26.2%) than among non-persistent one (53.9%), and affected 34.1% of patients in the overall study population.

### Impact of demographic and clinical characteristics on persistence

**Figure 1.**
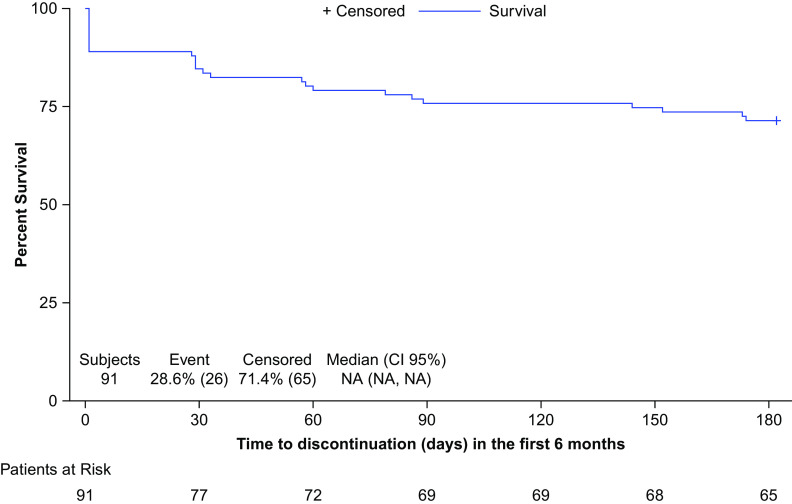
Time (days) to all cause treatment discontinuation in first six months (Kaplan–Meier).

Mean AOM persistence time during the first six months of treatment was 182.0 days in the persistent population and 48.5 days in the non-persistent. The mean (SD) time estimate to all cause treatment discontinuation in the first six months was 138.1 (6.8) days, with the majority of discontinuations occurring during the first 30 days (Figure 1).

Demographic and clinical characteristics that were associated with persistence on AOM at six months (univariate Cox regression analysis *p* < 0.15) were: Number of previous AP within the 2 years prior to the index date, Last AP prior to AOM: Olanzapine; Concomitant schizophrenia treatments at index date; History of non-adherence in the 3 months prior to the index date; Time since schizophrenia diagnosis; Reason to initiate AOM: Prevent relapse; Reason to initiate AOM: Prevent discontinuation; Reason to initiate AOM: Improve adherence to treatment; CGI-S at the index date and Maximum tolerated AOM dose. These variables were pre-selected to be included in the multivariate regression.

**Figure 2.**
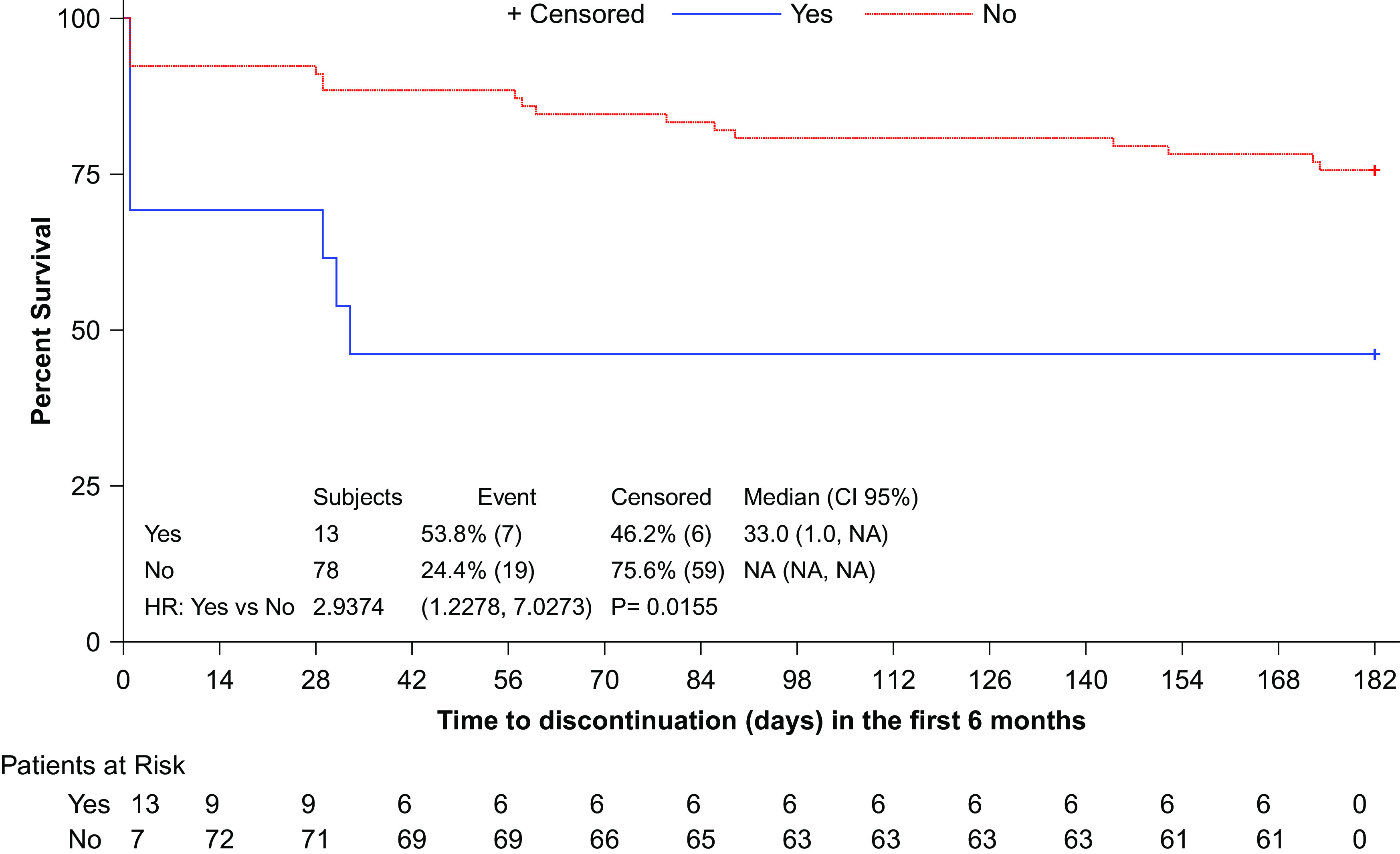
Time (days) to all—cause treatment discontinuation during the first six months of treatment (Kaplan–Meier). Patients were stratified by with the presence of concomitant schizophrenia treatments at index date.

Backward selection method was done until reaching a maximum level of 5% significance for the least significant variable. Two variables (Concomitant schizophrenia treatments at index date and Time since schizophrenia diagnosis) remained in the final (multivariate) Cox regression model; both fulfilled the proportional hazard assumption. In the final multivariate model, each one-year increase since schizophrenia diagnosis was associated with a 1.05-fold increase in the risk of AOM discontinuation in the first six months (HR: 1.05; 95% CI: 1.02–1.09; *p* = 0.003). Additionally, patients with concomitant schizophrenia treatments at index date were 2.86 times more likely to experience AOM discontinuation in the first six months than patients without them (HR: 2.86; 95% CI: 1.18–6.93; *p* = 0.02) (Figure 2).

### Treatment discontinuation

In the first six months, 26 patients (28.6%) discontinued AOM treatment. The main reasons for treatment discontinuation were adherence problems and psychotic relapse, with or without hospitalization, (23.1% each), followed by patient/family choice and discontinuation of patient’s visits (19.2% each) (Table 2).

**Table 2.** Treatment discontinuation rate and reasons for discontinuation in first six months.

	All population (*N* = 91)
Treatment discontinuation rate in first six months	26 (28.6%)
Reason for treatment discontinuation in first six months	
Adherence problems	6 (6.6%)
Psychotic relapse and/or hospitalization	6 (6.6%)
Patient/family choice	5 (5.5%)
Lack of functional benefits	1 (1.1%)
Tolerability problems/safety	1 (1.1%)
Persistence of residual psychotic symptoms	1 (1.1%)
Convenience	1 (1.1%)
Discontinuation of patient’s visits	5 (5.5%)

### Global clinical severity

**Figure 3.**
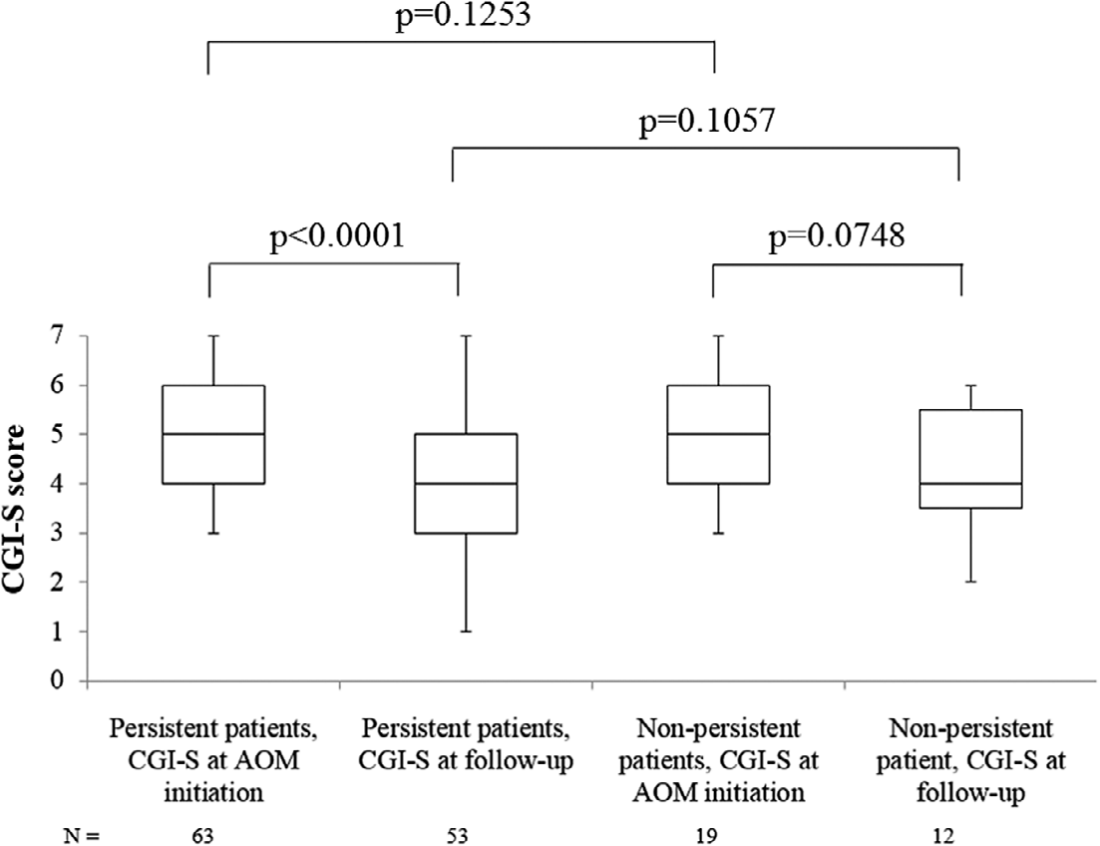
Performance of *Clinical global impressions-severity* (CGI-S) score.

At AOM initiation, patients were markedly ill (median CGI-S score of 5.0 [Q1, Q3: 4.0, 6.0]) whereas during the retrospective follow-up patients were moderately ill (4.0 [3.0, 5.0]). The median change in the CGI-S score between AOM initiation and follow-up was statistically significant in the persistent population only (−1.0 point [−2.0, 0.0]; *p* < 0.001), indicating a shift in symptom severity status (markedly ill to moderately ill) in these persistent patients. However, there were no statistical differences between subpopulations (Figure 3).

**Figure 4.**
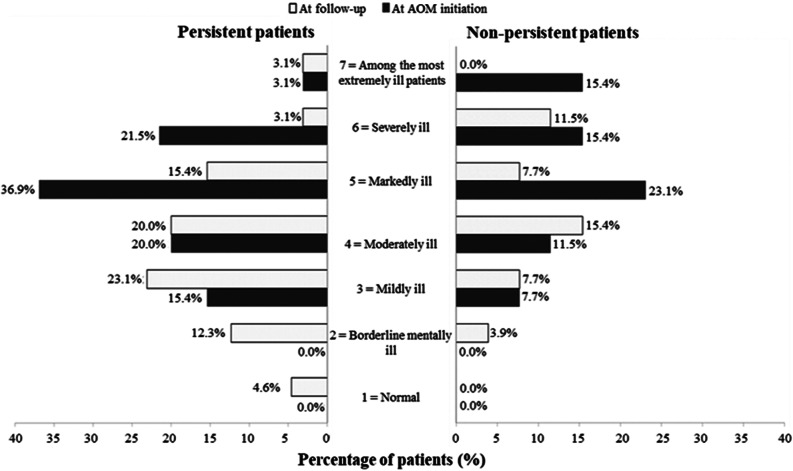
*Clinical global impressions-severity* (CGI-S) score frequencies at aripiprazole once-monthly (AOM) initiation and follow-up period.

At AOM initiation, 3.1% of patients in the persistent cohort were ranked as “among the most extremely ill”, 36.9% as “markedly ill”, and 21.5% as “severely ill” vs. 15.4, 23.1, and 15.4% of patients, respectively, in the non-persistent cohort. Six months after AOM initiation, percentage of “among the most extremely ill” patients in the persistent subgroup remained 3.1 vs. 0% in the non-persistent subgroup. However, during follow-up, percentages of “severely ill”, and “markedly ill” patients in the persistent subgroup decreased up to only 3.1 and 15.4%, respectively, in comparison to 7.7 and 11% of patients in the non-persistent population. Additionally, 12.3 and 4.6% of persistent patients were ranked as “Borderline mentally ill” and “normal” (CGI-S score of 2 and 1, respectively), at six months (Figure 4).

### Treatment patterns

During follow-up, a total of 4 (4.4%) patients (2 in each subpopulation) switched to an alternative schizophrenia medication. There was a total of 5 medication switching, being olanzapine (2, 3.1%) and zuclopenthixol (1, 1.5%) the switched to medications in the persistent population, and quetiapine (2, 7.7%) in non-persistent population.

In addition, 3 (3.3%) patients needed a medication augmentation after the AOM initiation: 2 (3.1%) in the persistent population and 1 (3.9%) in the non-persistent population. In the persistent population, the initiated treatment were olanzapine and quetiapine (1 patient, 1.5%, each) and in the non-persistent population was quetiapine (1, 3.9%). The mean time of overlap with AOM ranged from 53 to 53.5 days.

The median starting AOM dose was 400 mg in both subpopulations. Overall, only 15 patients were registered with dose changes, of which 12 were in the persistent population, mainly due to tolerability problems. None of them changed doses due to lack of efficacy.

### Psychotic symptoms

At AOM initiation, mean (SD) BPRS score was 50.1 (16.6), with no significant differences between subgroups (*n* = 36, *p* = 0.27). Six months after AOM initiation, mean BPRS score for persistent and non-persistent population showed a statistically significant decrease of 16.4 (*p* < 0.0001) and 13.7 (*p* = 0.0021) points, respectively. No statistical differences were found between the two subpopulations. Significant reductions in the BPRS positive symptoms score at follow-up for persistent (*p* < 0.0001) and non-persistent populations (*p* = 0.0024) were found, only statistical differences for BPRS negative symptoms score were found in the persistent population (*p* = 0.0004) (Table 3).

**Table 3.** Performance of Brief Psychiatric Rating Scale (BPRS).

		Persistence first six months: Yes (*N* = 65)	Persistence first six months: No (*N* = 26)	Total (*N* = 91)	
Total score					
AOM initiation	*n*	29	7	36	
	Mean (95%CI)	51.07 (44.18, 57.96)	46.29 (39.77, 52.80)	50.14 (44.53, 55.75)	
	Standard deviation (SD)	18.11	7.04	16.57	
	Median	51.00	47.00	48.50	
	(Q1, Q3)	(36.00, 66.00)	(42.00, 52.00)	(36.50, 63.00)	
	(Min, Max)	(22.00, 85.00)	(34.00, 55.00)	(22.00, 85.00)	
	*p*-value independent *T*-test (1)			0.2749	
Follow-up (retrospective period)	*n*	21	6	27	
	Mean (95%CI)	37.67 (31.31, 44.02)	31.67 (23.39, 39.95)	36.33 (31.20, 41.46)	
	Standard deviation (SD)	13.95	7.89	12.97	
	Median	36.00	31.00	36.00	
	(Q1, Q3)	(25.00, 51.00)	(25.00, 39.00)	(25.00, 46.00)	
	(Min, Max)	(19.00, 66.00)	(23.00, 41.00)	(19.00, 66.00)	
	*p*-value independent *T*-test (1)			0.3272	
Change: Follow up—AOM initiation	*n*	21	6	27	
	Mean (95%CI)	−16.43 (−21.20, −11.66)	−13.67 (−19.66, −7.67)	−15.81 (−19.61, −12.02)	
	Standard deviation (SD)	10.47	5.72	9.59	
	Median	−19.00	−14.50	−18.00	
	(Q1, Q3)	(−23.00, −11.00)	(−19.00, −9.00)	(−23.00, −10.00)	
	(Min, Max)	(−32.00, 4.00)	(−19.00, −6.00)	(−32.00, 4.00)	
	*p*-value Paired *T*-test (2)	<0.0001	0.0021	<0.0001	
	*p*-value Independent *T*-test (1)			0.5444	
Time (months) between BPRS assessment in AOM initiation and follow-up (retrospective period)	*n*	20	6	26	
	Mean (95%CI)	14.64 (11.00, 18.29)	21.25 (10.63, 31.87)	16.17 (12.68, 19.66)	
	Standard deviation (SD)	7.79	10.12	8.64	
	Median	15.62	23.59	16.39	
	(Q1, Q3)	(12.52, 17.26)	(20.44, 27.89)	(13.04, 20.04)	
	(Min, Max)	(0.99, 37.03)	(1.94, 30.06)	(0.99, 37.03)	
Positive symptoms score					
At AOM initiation	*n*	29	7	36	
	Mean (95%CI)	14.52 (12.49, 16.54)	12.43 (9.02, 15.84)	14.11 (12.40, 15.83)	
	Standard deviation (SD)	5.32	3.69	5.07	
	Median	16.00	14.00	14.00	
	(Q1, Q3)	(11.00, 18.00)	(9.00, 14.00)	(10.50, 18.00)	
	(Min, Max)	(4.00, 23.00)	(7.00, 18.00)	(4.00, 23.00)	
	*p*-value Independent *T*-test (1)			0.3351	
At follow-up (retrospective period)	*n*	21	6	27	
	Mean (95%CI)	9.05 (7.17, 10.92)	6.17 (4.49, 7.85)	8.41 (6.87, 9.94)	
	Standard deviation (SD)	4.12	1.60	3.88	
	Median	9.00	5.50	7.00	
	(Q1, Q3)	(5.00, 12.00)	(5.00, 7.00)	(5.00, 11.00)	
	(Min, Max)	(4.00, 17.00)	(5.00, 9.00)	(4.00, 17.00)	
	*p*-value Independent *T*-test (1)			0.0166	
Change: Follow up—AOM initiation	*n*	21	6	27	
	Mean (95%CI)	−5.62 (−6.89, −4.34)	−6.00 (−8.74, −3.26)	−5.70 (−6.78, −4.63)	
	Standard deviation (SD)	2.80	2.61	2.71	
	Median	−6.00	−6.50	−6.00	
	(Q1, Q3)	(−8.00, −4.00)	(−8.00, −4.00)	(−8.00, −4.00)	
	(Min, Max)	(−9.00, 0.00)	(−9.00, −2.00)	(−9.00, 0.00)	
	*p*-value Paired *T*-test (2)		0.0024		
	*p*-value Signed Rank (3)	<0.0001		<0.0001	
	*p*-value Wilcoxon (4)			0.8134	
Negative symptoms score					
At AOM initiation	*n*	29	7	36	
	Mean (95%CI)	9.86 (8.37, 11.35)	11.57 (8.37, 14.77)	10.19 (8.89, 11.50)	
	Standard deviation (SD)	3.92	3.46	3.85	
	Median	10.00	11.00	11.00	
	(Q1, Q3)	(6.00, 13.00)	(8.00, 14.00)	(6.50, 13.00)	
	(Min, Max)	(4.00, 17.00)	(7.00, 17.00)	(4.00, 17.00)	
	*p*-value Independent *T*-test (1)			0.2978	
At follow-up (retrospective period)	*n*	21	6	27	
	Mean (95%CI)	8.24 (7.03, 9.44)	9.67 (5.93, 13.40)	8.56 (7.42, 9.69)	
	Standard deviation (SD)	2.64	3.56	2.86	
	Median	8.00	9.50	8.00	
	(Q1, Q3)	(7.00, 10.00)	(7.00, 12.00)	(7.00, 10.00)	
	(Min, Max)	(4.00, 13.00)	(5.00, 15.00)	(4.00, 15.00)	
	*p*-value Independent *T*-test (1)			0.2893	
Follow up—AOM initiation	*n*	21	6	27	
	Mean (95%CI)	−2.76 (−4.13, −1.40)	−2.50 (−5.80, 0.80)	−2.70 (−3.88, −1.53)	
	Standard deviation (SD)	3.00	3.15	2.97	
	Median	−3.00	−1.50	−3.00	
	(Q1, Q3)	(−5.00, −1.00)	(−4.00, −1.00)	(−5.00, −1.00)	
	(Min, Max)	(−8.00, 4.00)	(−8.00, 1.00)	(−8.00, 4.00)	
	*p*-value Paired *T*-test (2)	0.0004	0.1092	<0.0001	
	*p*-value Independent *T*-test (1)			0.8533	

Abbreviation: AOM, aripiprazole once-monthly; BPRS, Brief Psychiatric Rating Scale; CI, confidence interval; SD, standard deviation.

(1) Independent Student’s t (parametric two independent samples T-test). Comparison between groups: Persistence Yes vs No.

(2) Paired Student’s t (parametric paired (samples) T-test). Comparison within group: Follow-up vs Initiation.

(3) Wilcoxon signed rank sum test (non-parametric analog to a paired samples T-test). Comparison within group: Follow-up vs Initiation.

(4) Wilcoxon-Mann-Whitney test (non-parametric analog to the independent samples T-test). Comparison between groups: Persistence Yes vs No.

### Patterns of drug abuse/dependence

At AOM initiation, the 29.2% of persistent patients and the 30.8% of non-persistent patients presented concomitant drug abuse or drug dependence. Most used drug was cannabis in both populations (24.6 and 23.1%). During the retrospective follow-up period, drug dependence/use was less frequent in both persistent and non-persistent population, however, drug consumption significantly decreased only in the persistent population (16.9%, *p* = 0.002 vs. 11.5%; *p* = not significant). The use of cannabis significantly declined from AOM initiation and during follow-up among persistent and non-persistent patients (15.4% vs. 11.5%; *p* = 0.002).

### Safety

There were 16 (17.6%) patients presenting a total of 26 AE/adverse drug reactions (ADR) during the retrospective follow-up period, being more frequent in the non-persistent population (*N* = 8, 30.8%; 16 AE/ADR) (Table 4).

**Table 4.** Adverse events (AE) and adverse drug reactions (ADR) reported during the retrospective follow-up.

	Persistence at first six months (*N* = 65)	Non-persistence at first six months (*N* = 26)	Total (*N* = 91)
Whole AE/ADR			
Patients with at least one AE/ADR, *n* (%)	8 (12.3%)	8 (30.8%)	16 (17.6%)
Total number of AEs/ADRs, *n*	10	16	26
Nausea	0 (0.0%)	1 (3.9%)	1 (1.1%)
Fatigue	0 (0.0%)	1 (3.9%)	1 (1.1%)
Gait disturbance	1 (1.5%)	0 (0.0%)	1 (1.1%)
Influenza	0 (0.0%)	1 (3.9%)	1 (1.1%)
Otitis externa	1 (1.5%)	0 (0.0%)	1 (1.1%)
Weight increased	1 (1.5%)	0 (0.0%)	1 (1.1%)
Hypercholesterolemia	0 (0.0%)	1 (3.9%)	1 (1.1%)
Pain in extremity	1 (1.5%)	0 (0.0%)	1 (1.1%)
Akathisia	0 (0.0%)	1 (3.9%)	1 (1.1%)
Bradykinesia	0 (0.0%)	1 (3.9%)	1 (1.1%)
Occipital neuralgia	0 (0.0%)	1 (3.9%)	1 (1.1%)
Restless legs syndrome	1 (1.5%)	0 (0.0%)	1 (1.1%)
Somnolence	1 (1.5%)	2 (7.7%)	3 (3.3%)
Syncope	0 (0.0%)	1 (3.9%)	1 (1.1%)
Tremor	2 (3.1%)	1 (3.9%)	3 (3.3%)
Insomnia	0 (0.0%)	1 (3.9%)	1 (1.1%)
Panic disorder	0 (0.0%)	1 (3.9%)	1 (1.1%)
Restlessness	0 (0.0%)	1 (3.9%)	1 (1.1%)
Hyperhidrosis	0 (0.0%)	1 (3.9%)	1 (1.1%)
Cyst removal	1 (1.5%)	0 (0.0%)	1 (1.1%)
Hypertension	0 (0.0%)	1 (3.9%)	1 (1.1%)
AE/ADR related to aripiprazole^a^			
Patients with at least one AE/ADR, *n* (%)	5 (7.7%)	2 (7.7%)	7 (7.7%)
Total number of AEs/ADRs, *n*	6	2	8
Gait disturbance	1 (1.5%)	0 (0.0%)	1 (1.1%)
Weight increased	1 (1.5%)	0 (0.0%)	1 (1.1%)
Akathisia	0 (0.0%)	1 (3.9%)	1 (1.1%)
Somnolence	1 (1.5%)	1 (3.9%)	2 (2.2%)
Tremor	2 (3.1%)	0 (0.0%)	2 (2.2%)


Data by preferred term shows number of patients with the AE/ADR at least once. One patient could suffer more than one AE/ADR.

Abbreviation: AE/ADR, adverse event/adverse drug reaction.

a Related to aripiprazole once-monthly (Abilify Maintena®) or aripiprazole (ONLY Abilify®brand).

A total of 8AE/ADR were treatment-related occurring in 7 (7.7%) patients, of which 5 (7.7%) occurred in patients from the persistent population (3 ADR were nervous system disorders), and 2 (7.7%) in patients from the non-persistent population. Only 1 patient (1.1%) presented akathisia, whereas 2 patients (2.2%) presented tremor, and 1 patient (1.1%) presented weight increase (Table 4).

In 2 (2.2%) patients these AE/ADR led to drug permanent withdrawal; 1 (1.5%) patient in the persistent population due to somnolence and 1 (3.9%) patient in the non-persistent population due to nausea. There were no serious AE/ADRs.

## Discussion

Poor adherence to AP treatment is related to worse disease prognosis, longer time needed to achieve remission and increased risk of relapse [31,34], hence, clinical efforts to improve adherence and treatment persistence is of capital importance in the management of schizophrenia.

In the last years, the use of LAI APs as a mean to improve medication adherence has been reflected in clinical guidelines [38], and prior studies have pointed to a higher capacity of LAIs compared to oral APs in relapse prevention [23].

PROSIGO is the first observational, retrospective, non-interventional study describing the impact of patients’ demographic and clinical characteristics on AOM treatment persistence, exclusively conducted in adult patients with schizophrenia who initiated AOM as primary maintenance AP treatment after being stabilized from an acute relapse, and before being discharged from an inpatient setting.

The 71.4% AOM persistence rate during the first six months of treatment in our study is consistent with AOM registration clinical trials rates of 74.7% [44] and 75.1% [40]. Though lower than the 86% persistence rate observed in DOMINO study [49], and 84% adherence rate reported in a German non-interventional AOM study [53], PROSIGO stay-on-treatment rate is in line with other AOM naturalistic studies [45,50,54], and greater than those reported for other LAI APs [45,55–57]. Only 28.6% of patients discontinued AOM during the first six months of treatment; 6 patients due to adherence problems, 6 patients due to psychotic relapse and/or hospitalization, and 6 patients due to patient/family choice.

Previous naturalistic studies have subscribed the efficacy of AOM as maintenance treatment of schizophrenia [45,53,54], and our results provide further data supporting significant improvements in psychopathology, as measured by CGI-S and BPRS scores, in persistent patients at six months after treatment initiation.

The median time estimate to all-cause treatment discontinuation in the first six months could not be calculated in the Kaplan–Meier due to the low number of events. The estimation of mean (SD) was 138.1 (6.8) days, with the majority of discontinuations occurring during the first 28 days.

We were able to describe several predictive factors for treatment persistence with AOM in our study population. Results from the multivariate Cox regression model showed that each increase in 1 year since schizophrenia diagnosis was associated with a 1.05-fold increase in the risk of AOM discontinuation in the first 6 onths (HR: 1.05; 95% CI: 1.02–1.09; *p* = 0.003), and that patients with concomitant schizophrenia treatments at index date were 2.86 times more likely to experience AOM discontinuation in the first six months than patients without concomitant schizophrenia treatments at index date (HR: 2.86; 95% CI: 1.18–6.93; *p* = 0.02). The incremental risk of discontinuation among patients with longer time since diagnosis could be triggered by different reasons such as chronic course of illness where repeated psychotic relapses might have severely impaired patients’ capacity to respond to pharmacological interventions, functionality, brain plasticity and receptor hypersensitization, or worsened insight [35,58,59], all features contributing to a less favorable profile for AP treatment in general.

Despite a better performance of AOM treatment in patients early in the disease, in terms of symptomatology and quality of life improvement, was already observed in a by-age sub-analysis of QUALIFY study, where patients ≤35 years obtained significantly greater benefits from AOM treatment than population >35 years old [45], we were not able to find statistical differences in persistence with AOM treatment in our study population when patients were sub-analyzed by the same cut-off age (*p*-value = 0.2456). This could be due to the non-interventional, retrospective design of the study and to the low number of patients included in the final analysis.

The relationship between AP polypharmacy and discontinuation observed in our study has also been noted in a previous real-world study retrieving data from a nationwide database, in which patients with schizophrenia receiving 2 second-generation APs presented a shorter median time to all-cause discontinuation compared to patients receiving these agents in monotherapy [60].

Regarding substance dependence/abuse, the most frequently used drug in both subpopulations was cannabis, in line with the results of a recent survey conducted among psychiatrists attending psychotic patients with concomitant substance use [61]. Both persistent and non-persistent patients experienced a decrease in cannabis consumption during the follow-up period that may have been the main driver for the decrease seen in both groups (significant for persistent patients) in overall drug dependence/abuse. Other recent studies support AOM as an efficacious treatment for patients diagnosed with schizophrenia with coexisting substance use; for example, an Italian prospective trial in psychotic patients reported a significant reduction in patient-reported substance craving after 1 year of AOM treatment [62] and an Spanish multicenter, observational, retrospective study suggested that AOM treatment retains its AP efficacy in patients with schizophrenia and a coexisting substance abuse disorder and could be useful for cocaine or alcohol use disorders management [63].

AOM showed a favorable tolerability profile with 7 (7.7%) patients presenting AEs related to AOM, rates of akathisia (1.1%), tremor (2.2%), and weight gain (1.1%) were low. Other previously ADRs reported with AOM (insomnia and injection pain) were not found in our study. In this regard, previous prospective studies [64,65] and an expert survey [39] pointed that between 35 and 50% of patients with schizophrenia found drug-related AE as deterrent factor and a handicap for treatment adherence. However, other important factors highlighted in PROSIGO, such as patients’ perceptions of medications effectiveness (closely connected to patients’ preference), have been identified as main contributors to adherence problems. It is important to note that several guidelines consider the “preference by the patient” as a reason to initiate LAIs at any illness stage [66–69].

The study presents several limitations due to its naturalistic, non-interventional design. First, patients’ management was in accordance to regular Spanish clinical practice, and patient’s willingness to participate in the study could imply a selection bias and a limited generalization of PROSIGO results to different healthcare systems. Second, the limited sample size and the retrospective nature of information was considered when available in the patients’ records, and even though patients with at least 10% of baseline clinical and demographic variables available were included, other important factors may have been missed. Third, there are no other reference points than index date, this together with a lack of randomization could mask other non-identified possible confounder factors.

Our results suggest that the main factors predicting persistence with AOM treatment at six months are fewer years since schizophrenia diagnosis and not receiving concomitant schizophrenia treatments at AOM initiation. An early initiation of AOM treatment would expand its persistence, which in turn is associated with decreased use of concomitant psychiatric treatments. In addition, the study shows that AOM treatment improved CGI and BPRS scores, coupled with a favorable safety profile.

## Data Availability

The data that support the findings of this study are available from Otsuka–Lundbeck. Restrictions apply to the availability of these data, which were used under license for this study. Data could be available with the permission of Otsuka–Lundbeck.
