# Predictors of persistence in patients with schizophrenia treated with aripiprazole once-monthly long-acting injection in the Spanish clinical practice: a retrospective, observational study – CORRIGENDUM

**DOI:** 10.1192/j.eurpsy.2021.2238

**Published:** 2021-10-11

**Authors:** José Manuel Olivares, Ana González-Pinto, Mario Páramo

**Keywords:** schizophrenia, persistence, aripiprazole, antipsychotic, predictors, corrigendum

This article when published failed to include the full list of Dr Ana González-Pinto’s affiliations, which are as follows:

- Hospital Universitario de Álava, Álava, Spain
- CIBERSAM, Biomedical Research Centre in Mental Health Network, Madrid, Spain
- Universidad del País Vasco, Álava, Spain
- IIS Bioaraba, Álava, Spain
